# Ngalaiya Boorai Gabara Budbut: A Qualitative Study With Primary Care Providers to Understand Perceived Needs, Enablers, Barriers and Opportunities to Strengthen Care

**DOI:** 10.5694/mja2.70150

**Published:** 2026-03-04

**Authors:** Christianna Digenis, Rachel Reilly, Peter Azzopardi, Hilina Winkenweder, Odette Pearson, Kane Ellis, Jane Fisher, Debra J. Rickwood, Choong‐Siew Yong, Ngiare Brown

**Affiliations:** ^1^ Wardliparingga Aboriginal Health Equity, South Australian Health and Medical Research Institute Adelaide South Australia Australia; ^2^ Adelaide University Adelaide South Australia Australia; ^3^ Burnet Institute Melbourne Victoria Australia; ^4^ Illawarra Aboriginal Medical Service Wollongong New South Wales Australia; ^5^ Monash University Melbourne Victoria Australia; ^6^ University of Canberra Canberra Australian Capital Territory Australia; ^7^ Headspace National Youth Mental Health Foundation Ltd Melbourne Victoria Australia; ^8^ Northern Sydney Local Health District Sydney New South Wales Australia; ^9^ Djanaba Child and Adolescent Centre of Excellence, Ngaoara Limited Wollongong New South Wales Australia

**Keywords:** adolescence, primary health care, social determinants of health

## Abstract

**Objective:**

To explore primary care providers' perspectives on (i) healthcare needs and barriers to care for Aboriginal and Torres Strait Islander children and adolescents; and (ii) enablers and opportunities to strengthen care.

**Study Design:**

A qualitative study; interviews and open‐ended survey responses.

**Setting:**

Primary care providers who work with Aboriginal and Torres Strait Islander children and young people in health and education settings in New South Wales and the Australian Capital Territory. Data were collected between 23 March 2022 and 13 October 2023.

**Main Outcome Measures:**

Sixteen interviews and 33 open‐ended survey responses were analysed using reflexive thematic analysis with a hybrid inductive/deductive approach.

**Results:**

Participants reported that some of the most important health needs for children and young people related to mental health. They recognised that the presenting complaint was not always the underlying or only concern, demonstrated an understanding of trauma‐informed care and acknowledged the importance of collaborative services that engaged support networks. Barriers to care included a lack of cultural safety in mainstream services, challenging social circumstances, financial concerns, being unaware of available services and privacy and confidentiality concerns. To improve care, staff identified several areas needing change including having a package of services tailored for young people; additional training for providers in child and adolescent health, particularly for mental healthcare, trauma‐informed care and communication with young people; providing a safe and engaging environment; support for staff self‐care; and additional resources.

**Conclusions:**

Supporting mental health needs is a key aspect of caring for children and adolescents. To provide optimal primary healthcare, service providers require specialist skills. To support adolescents and children, participants identified a need for ongoing training, professional development and organisational support to ensure best practice care is sustained. This work has informed the development of training and other resources for partner health services.

## Plain Language Summary

**The known:** Childhood and adolescence are key developmental windows during which distinct, though often under‐recognised, healthcare needs can emerge. For Aboriginal and Torres Strait Islander children and young people, many health needs are not met by existing policies and services.

**The new:** Primary healthcare service providers have identified roadblocks in the way care is delivered to children and adolescents, and the importance of improving the way key priorities, such as mental health, are addressed.

**The implications:** Improving primary healthcare for Aboriginal and Torres Strait Islander children and adolescents has implications for health now and into the future. Focussing service improvements around social and emotional well‐being, cultural safety and trauma‐informed care remains the key priority.

## 1 Introduction

Over half of the Aboriginal and Torres Strait Islander population in Australia is under the age of 24 years [1, 2]. Primary healthcare can play a key role in disease prevention and promotion of health and can reduce the burden on secondary and tertiary services. This is particularly true when primary care is linked with secondary and community services [3, 4]. This has been recognised in the current *National Aboriginal and Torres Strait Islander Health Plan (2021–2031)* [5] and the *National Strategic Framework for Aboriginal and Torres Strait Islander Peoples' Mental Health and Social and Emotional Wellbeing* [6].

However, primary healthcare services often work in an environment characterised by high demand for acute needs and limited financial and human resources. In this context, provision of high‐quality care to individuals with high or complex needs—including mental health concerns—can be challenging. Our earlier work has highlighted that Aboriginal and Torres Strait Islander children with mental health support needs, especially those that stem from the experience of complex trauma, do make contact with primary care, but their needs are not always met [7]. As such, there are opportunities to strengthen primary healthcare, particularly through the Aboriginal Community Controlled Health Services (ACCHS) sector, which is best placed to provide culturally safe and trauma‐informed care.

The World Health Organization (WHO) outlines eight key standards to ensure responsive primary healthcare for adolescents [8], which incorporate both ‘supply’ (service‐related) and ‘demand’ (client/community‐related) factors (Table 1). An additional ninth standard regarding cultural safety has also been suggested [9, 10] (Table 1). The current evidence on mental healthcare for Indigenous youth globally indicates a need for greater focus on supply factors, for example, building provider competencies in child and adolescent health, offering youth‐relevant services and providing culturally safe, equitable and non‐discriminatory care [9]. Previous work has found gaps in appropriate screening tools, guidelines and additional training to support young peoples' mental healthcare needs [11]. Given the focus on supply factors in this context, it is critical to understand the views of providers on delivering care and understand areas of improvement at both individual and systemic levels, including training and resource needs [12].

**TABLE 1:** Adapted World Health Organization (2015) Global Standards for Quality Health‐Care Services for Adolescents [8].

Standard	Demand/supply?
Standard 1: *Health literacy*: The health service implements systems to ensure adolescents are knowledgeable about their own health, and they are aware of where and when to obtain health services	Demand
Standard 2: *Community support*: The health service implements systems to ensure that parents, caregivers, community members and organisations recognise the importance of delivering health services to adolescents. The community supports access and utilisation of the services by adolescents	Demand
Standard 3: *Appropriate package of services*: The health service provides a package of information, counselling, diagnostic, treatment and care services that addresses the needs of all adolescents. Such services are provided in the facility/health service and through referral pathways and outreach	Supply
Standard 4: *Providers' competencies*: Healthcare providers demonstrate the required competences to provide effective care to adolescents. Adolescents' rights to information, privacy, confidentiality, non‐discrimination, non‐judgemental care and respect are protected and provided by healthcare and support staff	Supply
Standard 5: *Facility characteristics*: The health service has convenient opening hours, a welcoming and clean environment and maintains the privacy and confidentiality of adolescents. The service has equipment, medicines, supplies and technology available to ensure effective service delivery to adolescents	Supply
Standard 6: *Equity and non‐discrimination*: The health service provides quality services to all adolescents irrespective of their ability to pay, age, sex, marital status, education level, ethnic origin, sexual orientation or other characteristics	Supply
Standard 7: Data and quality improvement: The health service collects (disaggregated by age and sex), analyses and uses data on adolescents' service use and measures quality of care, to support quality improvement. Staff members are supported to participate in continuous quality improvement	Demand
Standard 8: *Adolescent participation*: Adolescents are involved in the planning, monitoring and evaluation of the health services and in certain appropriate aspects of service provision. Adolescents are also involved in decisions regarding their own care	Demand
Additional Standard 9: *Cultural safety* [9, 10]: Adolescents are provided culturally safe care, which reflects their own culture and practices. This includes language, traditional healing and medicine, cultural protocols, the presence and involvement of Indigenous healthcare providers in the delivery of care. As well as the acknowledgement of the historical context of colonisation and racism and how this correlates with health and well‐being and the delivery of care	Supply

To improve responsiveness to mental health needs in primary healthcare, a larger mixed‐methods project entitled *Ngalaiya Boorai Gabara Budbut* (*Supporting the heads and hearts of Aboriginal and Torres Strait Islander Children and Adolescents*) was established in collaboration with five health and/or education services in regional and remote New South Wales and the Australian Capital Territory, which provide healthcare and support to Aboriginal and Torres Strait Islander young people and their families. The larger study includes interviews with children and their caregivers, and the development of culturally appropriate assessments tools and resources [12]. This study aims to understand providers' perspectives on (i) healthcare needs and barriers to care for Aboriginal and Torres Strait Islander children and adolescents; and (ii) enablers and opportunities to strengthen care for Aboriginal and Torres Strait Islander children and adolescents.

## 2 Methods

This pragmatic exploratory qualitative study included open‐ended responses to a survey and in‐depth interviews with service providers. The project was guided by the Consolidated Framework for Implementation Research [13, 14], which has been widely used in Indigenous research as it aligns with decolonising principles that privilege Indigenous voices [14].

The online staff survey was implemented in two partner organisations between 23 March and 12 September 2022 and an additional two organisations between 12 September 2023 and 13 October 2023 [5]. Dates for data collection were chosen by the organisations. Participants responded to the survey via a link emailed from organisational management. Only qualitative responses to open‐ended questions are included in this analysis. The survey questions are provided in the Supporting Information. Quantitative data were provided to health services for continuous quality improvement purposes but are not included here.

Semi‐structured interviews exploring experiences and perspectives on providing care to Aboriginal and Torres Strait Islander children and adolescents were conducted with service providers from the five organisations. These included the four original services from the survey as well as an educational support service. Interviews were conducted between March 2022 and August 2023 until there was representation from each partner service. Interviewees were recruited via professional networks and snowball sampling within organisations. Interviews were conducted in person in a private location, by telephone or online, according to the preference of the interviewees. Interviews were audio‐recorded and transcribed for analysis. The interview questions are provided in the Supporting Information. Interview transcripts and open‐ended survey questions were imported into NVivo software and analysed together using reflexive thematic analysis following a hybrid inductive/deductive approach [15, 16, 17]. Data were categorised into needs, barriers and enablers/opportunities and themes within those categories were described inductively. Themes were then reviewed according to their alignment with the WHO Global Standards for Quality Health‐Care Services for Adolescents (Table 1) [8].

## 3 Research Team, Governance and Reflexivity

Interviews were conducted by four non‐Indigenous researchers who were psychologists and/or experienced qualitative researchers who work in Aboriginal and Torres Strait Islander health and/or research. All interviews were analysed by the lead author, and a subset was coded by three other members of the research team. Any differences in coding were discussed and resolved without need for additional review. The team met with the principal researcher (NB), a senior Aboriginal medical practitioner, to discuss findings to ensure relevance and appropriateness.

Following reflexive practice and protocols of reciprocity, early findings of themes were presented to staff at one of the participating services and regularly reported to and discussed with the governance group, which consisted of representatives from the partner services, a youth representative and the investigator group. Themes and supporting quotes reflect the six‐phase process outlined by Braun and Clarke [16]. At completion of the overarching project [12], a community report will be produced and disseminated, and presentations at participating services are planned.

## 4 Bias/Ethical Considerations

This study was approved by the Aboriginal Health and Medical Research Council (AHMRC) of the New South Wales Human Research Ethics Committee (1769/21) and was conducted in line with the South Australian Aboriginal Health Research Accord [18]. The article is written in accordance with the COnsolidated criteria for REporting Qualitative research (COREQ) checklist [19] (Supporting Information), the CREATE Quality Appraisal Tool [20] and the CONSolIDated critERia for strengthening reporting of health research involving Indigenous peoples (the CONSIDER statement) checklist [21] (Supporting Information). Participation was voluntary and participants were provided with an information sheet and consent form outlining the study. Data were de‐identified before analysis and were stored securely and confidentially.

## 5 Results

### 5.1 Participants

The survey participants comprised 32 service providers with a mean age of 40 years. Gender was asked as an open‐ended field, and most participants identified as female (78%), 5 as male, and 3 left the field blank. All participants had contact with children or young people as part of their work role. Participants' roles included nursing (12), general practice (3), allied health (13) and ‘other’ (4), with an average of 3 years in their current roles. Seven (22%) respondents reported being Aboriginal and/or Torres Strait Islander. Between the four organisations who participated in the survey, about 100 staff were eligible, indicating a response rate of 33%.

The interviewees comprised 16 service providers, of whom nine (56%) were Aboriginal and/or Torres Strait Islander. Participants' roles included educational support (4), psychology (2), general practice (2), senior management (4), nursing (2), Aboriginal health practice (1) and social work (1) The interview duration was about 40 min.

#### 5.1.1 Theme 1: Perceived Needs of Children and Young People Presenting to Primary Healthcare

##### 5.1.1.1 Priority Health Issues for Young People Span Both Physical and Mental Health

Participants described mental health concerns stemming from trauma, the effects of bullying and racism, alcohol and other drugs and behavioural challenges as the most commonly and consistently reported health issues for young people. Sexual health alongside chronic diseases; ear, nose and throat conditions; general physical health and dental health were also mentioned.

… Apart from their little, you know, ear infections …, if it's not pregnancy or like [an] STI [sexually transmitted infection] checkup (that would be like about 25% …) is mostly mental health issues. (Interview 1: general practitioner)

… Primary presenting issues are around symptoms I would associate with developmental trauma. …. a level of generational trauma as well. (Interview 14: senior manager)

##### 5.1.1.2 The Presenting Issue Is Not Always the Key Issue

Service providers recognised that the issues outlined above were not always the first ones mentioned when children and adolescents presented at their appointment. The importance of asking questions ‘beyond the presenting complaint’ to enable discussion about areas of concern including home life, school and social and emotional well‐being was recognised.

… [Teenagers] might be coming in … with like a cold or flu. And then our GPs [general practitioners] are the ones recognising the age and being like, “Okay, let's have a chat about …” like sexual health, mental health, that sort of thing. (Interview 2: nurse)

##### 5.1.1.3 Understanding and Responding to Complex Trauma

A trauma‐informed care approach was considered critical to providing appropriate care to young people, as providers believed that complex trauma was a key driver of many of the mental health presentations of children and adolescence. To staff, trauma‐informed care meant understanding the young person's experiences (such as generational trauma, adverse childhood experiences and bullying), how these experiences impact on all aspects of the young person, and how they present in practice. While staff demonstrated an understanding of trauma‐informed care concepts, some reported that they were not confident providing mental healthcare, including addressing issues such as trauma (Table 1, Standard 4: Providers' competencies).

To provide “trauma‐informed care” you must have an understanding of how trauma can affect young people's lives. This includes behaviourally, physiologically and mentally. (survey response: allied health)

Complex trauma was also discussed as a suggested area of additional training (see below).

#### 5.1.2 Theme 2: Barriers to Young People Accessing Healthcare

##### 5.1.2.1 Awareness of Health Systems, Services and Conditions

Interview participants suggested that culturally appropriate referral options for young people with mental health concerns were limited, with long wait times. Simultaneously, a lack of awareness among young people and their support networks about health systems, services and health conditions was identified as a barrier to accessing healthcare (Table 1, Standard 1: Health literacy); highlighting the need for primary healthcare to better support their awareness and understanding.

A lot of mob feel embarrassed to admit their lack of understanding … leave [appointment] without having a complete understanding of their condition and/or treatment plan. (survey response: other)

This was particularly true for children and adolescents without family support. Despite having the right to attend independently, without such family support, they are unlikely to engage.

… Health literature [to understand] … the age you can get your own Medicare card. (Interview 3: Aboriginal health practitioner)

##### 5.1.2.2 Culturally Safe Systems, Schools and Healthcare Are Critical to Access

Experiences of unsafe care, including racism, in the wider system, especially mainstream services (education sectors and healthcare) were barriers for young people to thrive; including in their healthcare access (Table 1, Additional Standard 9: Cultural safety). In addition, participants reported a need for resources and guidelines regarding culturally safe ways of working for both Aboriginal and Torres Strait Islander and non‐Indigenous staff, suggesting that such knowledge should not be assumed, and learning can be ongoing, even for those who are proficient:

Systemic racism in mainstream hospitals … It's a huge barrier … because no one feels comfortable going to hospital in the first place, none of it's culturally appropriate. (Interview 2: nurse)

Includes racism and its impacts. The lack of culturally safe care in mainstream services and for services to be Aboriginal led and open to learning from Elders. (Interview 7: senior manager)

Service providers identified that trust, rapport and culturally appropriate care between young people, their support networks and providers supported engagement in healthcare, by helping young people feel safe (Table 1, Standard 4: Providers' competencies; Additional Standard 9: Cultural safety).

… Culture‐wise …. mainstream [people] here [at the medical service] need the protocols and need to learn about that [culture] as well and understand who we [Indigenous people] really are (Interview 12: nurse)

Some participants described the benefit of young people knowing their provider.

… A regular health professional is really, really important … person that they trust … (Interview 1: general practitioner)

##### 5.1.2.3 Socio‐Economic Barriers to Accessing Best Practice Healthcare

Cost, limited access to bulk‐billing services and issues with transport and housing were identified as barriers to healthcare access for some young people. Few staff reported knowledge of free or affordable service options for adolescents (Table 1, Standard 5: Facility characteristics; Standard 6: Equity and non‐discrimination).

The dietician would give them a sheet [list of foods] … I just don't think they could afford it … (Interview 13: general practitioner)

… I know lots of families struggle financially, so there's things like petrol, affording a car … so then kids can't come to appointments. … There's a lot of sickness and that's because they don't have regular health checks or immunisations … (Interview 15: social worker)

##### 5.1.2.4 Having Appropriate Resourcing

In some services, a lack of resources was identified as a barrier to service providers providing high‐quality care. This included enough time to spend with patients, appropriate and timely external referral options and consistent staff to deliver care (Table 1, Standard 3: Appropriate package of services).

GPs are overrun themselves … and you can't get a referral, … there's no one sitting in that chair … (Interview 8: educational support worker)

#### 5.1.3 Theme 3: Enablers and Opportunities to Improve Care

##### 5.1.3.1 Providing Flexibility in Care

Service providers suggested a flexible service model, particularly for mental healthcare. Suggestions varied from having access to multiple services and health specialists in one location, flexible opening times and short waiting times, providing transport and for health services to be in convenient, accessible locations, for example, close to public transport or in the community (school, community centres, in‐home) (Table 1, Standard 5: Facility characteristics).

Even if it was not an everyday service just like a bus … access on‐site to whatever they may need … just have the ability to do everything on the day. (Interview 4: senior manager)

… We need to be more mobile in community. I think we need to get away from … the four walls and a roof [clinic] and expect everyone to come to us … (Interview 7: senior manager)

Others suggested having the option for telehealth to improve access for young people.

[Facilitate] proper video, telehealth … (Interview 1: general practitioner)

##### 5.1.3.2 Creating a Friendly and Engaging Space for Young People

The physical space of the health service was considered crucial for engaging young people. Making the visual and physical spaces more culturally and age appropriate, and less clinical, for example, by adding toys and colours, was thought to improve engagement (Table 1, Standard 5: Facility characteristics).

[Rooms] can be quite clinical at times … [rooms] could certainly be changed to create a more positive environment. Having artwork or a bit of colour … (Interview 5: psychologist)

Another provider from a different service reflected:

It's a beautiful clinic with lots of sensory things and toys and colouring, … healthy snacks … (Interview 15: social worker)

To better engage with young people, a good strategy to consider was the health service being a space not just for healthcare but also a place where young people can relax, have a snack or do homework.

You are welcome if you do not have an appointment, you don't actually have to see someone … had kids just drop in [health service] … to do some homework … (Interview 15: social worker)

##### 5.1.3.3 Additional Training and Skills

Participants expressed a high level of interest in training to build skills in working with children and adolescents, including ‘… upskilling [general practitioners] about mental health’ (Interview 5: psychologist). Others identified that training in complex trauma ‘… could be a useful tool’ (Interview 6: nurse). A need to have access to training in assessment tools and processes, and better communication with Aboriginal and Torres Strait Islander young people, was also highlighted.

Training … around your assessments of young people … how to communicate with a young girl or a young boy. (Interview 3: Aboriginal health practitioner)

Service providers also identified that while knowledge of cultural protocols was often assumed given the nature of the participating services, this was not always the case.

What they really need here, is mainstream people need to know the protocols of Indigenous cultural ways (Interview 12: nurse)

##### 5.1.3.4 Incorporating Young People's Support Systems

Most service providers identified that providing high‐quality care for young people involved working with their support networks, including their caregivers/parent, grandparents, Elders, school and their broader communities (Table 1, Standard 2: Community support). Care was enabled by communicating with and supporting the family and caregivers of the young person.

I guess I look at young people and teenagers as a whole unit with their parents or with their carers (Interview 1: general practitioner)

Service providers found that parents' or caregivers' engagement with the healthcare service supported young people's engagement either practically, by bringing them to the health service and/or by modelling healthy behaviours and help seeking.

In a way that when you connect to the parents, the kids will follow (Interview 1: general practitioner)

But this needed to be balanced with respect for privacy and confidentiality (Table 1, Standard 4: Providers' competencies; Standard 5: Facility characteristics).

They're too scared to go to the doctor because [the young person thinks] it's going to go back to their parents because they're on their Medicare card. (Interview 3: Aboriginal health practitioner)

This could extend to concerns that caregivers or others within the community could access their health records and judge them negatively.

“Everyone's going to see your records, everyone's going to talk about why you're there”, so we need to change that mindset (Interview 7: senior manager)

##### 5.1.3.5 Holistic Care Is Multidisciplinary and Cross‐Sectorial

Most service providers identified that a multidisciplinary and multi‐systems (community services, education, justice, child protection) approach to wrap around young people with appropriate care planning was critical (Table 1, Standard 3: Appropriate package of services). This includes enabling access to multiple specialist service providers, for example, medical, psychological and other allied health and working with key community organisations, especially schools.

I think a big difference here is that you have access to a multidisciplinary team (Interview 16: psychologist)

It's just trying to work with the schools around that and providing better support (Interview 7: senior manager)

Providers acknowledged challenges delivering this type of care, including services working in silos, issues with caseloads or challenges with communication. In addition, participants' knowledge of external resource availability (e.g., referrals, guidelines) was generally low.

Services need to collaborate together, … it can't be individual … all of them need to work together (Interview 1: general practitioner)

##### 5.1.3.6 Supporting Healthcare Provider Self‐Care

Service providers who had experience working with complex trauma acknowledged the impact of vicarious trauma and burn‐out. This affected their well‐being and ability to work with complex trauma and young people. Concerns around complexity and working with young people may also impact on service providers' desire to work in this context.

I've worked with complex children for 12 years, I just think nobody can see that many kids and that many complex kids all day … (Interview 14: senior manager)

If staff burn‐out … they leave (Interview 15: social worker)

## 6 Discussion

Aboriginal and Torres Strait Islander young people have a high level of unmet mental and physical healthcare needs [22], which require more responsive healthcare services. Although leaders in providing comprehensive, culturally safe, trauma‐informed primary healthcare across the life course [23], ACCHS themselves identified that there may be opportunities to improve care. This project responds to calls from four ACCHS for research to understand how they can better meet the needs of Aboriginal and Torres Strait Islander children and adolescents [12]. Our findings align with national data on the well‐being of Aboriginal and Torres Strait Islander children, which show that mental health, including trauma, are key priorities in primary healthcare for children and adolescents [22, 24].

Our findings suggest a need to strengthen expertise through additional training for mental health, social and emotional well‐being and trauma‐informed care. This is consistent with previous research that found training, resources and professional development to improve practitioner competence and self‐efficacy may help to improve care to children and young people [25, 26]. Increasing knowledge through continued professional development is feasible as it is not resource intensive to health services.

Staff also identified several strategies to support care. They highlighted the benefits of building relationships and working in a collaborative way with external organisations, family and community. Improving communication skills was considered important for improving access for young people and their support networks, as it would assist in facilitating health literacy. This is also consistent with previous work that identified that a collaborative care approach supported access for young people [27] and is an area where services (educational, community and health) can improve.

This work supports the notion that standards of care should include cultural safety for Aboriginal and Torres Strait Islander young people. Barriers, enablers and opportunities identified by service providers aligned with most of the WHO standards, aside from Standard 7: Data and quality and Standard 8: Adolescent participation. Importantly, Harfield and colleagues [9] reported that self‐determination, empowerment and inclusion in decision‐making were key enablers for adolescents' mental healthcare access. Of note, in our research, adolescents' inclusion in decision‐making did not emerge as a prominent theme (Table 2), pointing to a service gap or potential focus for future work. Overall, our findings point to several ways that primary health services are meeting the needs of Aboriginal and Torres Strait Islander children and adolescents and suggest that appropriate resourcing to address under‐staffing enables ACCHS to deliver the care they are expert in. Such resourcing issues can compound disadvantages, which impacts on the health and well‐being of Aboriginal and Torres Strait Islander Peoples [28]. Larger systemic barriers to care, such as a lack of available mental health referral options, require a broader policy response.

**TABLE 2:** Barriers, enablers and opportunities aligning with the adapted World Health Organization (2015) Global Standards for Quality Health‐Care Services for Adolescents [8].

Standard	Health service characteristic identified in the data
1. Health literacy	Awareness among young people and their support networks about health systems, services and health condition Services and providers need to better communicate and ensure understanding of health conditions and awareness of available services
2. Community support	The young person's support networks are crucial in supporting accessing to healthcare Engaging support networks in care – Elders, school, broader community
3. Appropriate package of services	Resourcing: enough time to spend with patients, appropriate and timely external referral options and consistent staff to deliver care High‐quality care planning
4: Providers' competencies	Increase skills and confidence in trauma‐informed care Skills in establishing rapport, communication Privacy, confidentiality
5. Facility characteristics	Flexible service model Low cost Privacy, confidentiality Accessible, transport available Welcoming physical space, less clinical
6. Equity and non‐discrimination	Low/no cost
7. Data and quality	
8. Adolescent participation	
Additional Standard 9. Cultural safety [9, 10]	Culturally unsafe care is a barrier to young people thriving Aboriginal and Torres Strait Islander staff are needed Training in cultural protocols and culturally safe care is an ongoing requirement for all staff

The research points to several areas of health translation. In response to the findings from this work, a training package on child and adolescent mental healthcare, including complex trauma, has been developed and is ready for piloting [29]. The package can also be adapted for use with caregivers to increase health literacy. In addition to this, the broader project has the potential to improve resources for primary healthcare and could be used to strengthen current guidelines for health service providers. Future research could also seek to address the study aims in other jurisdictions across Australia.

## 7 Limitations

The study sample was small and lacking strong representation from medical staff (e.g., general practitioners); however, this limitation is mitigated by the qualitative methods employed. As the survey was anonymous and sent to all staff, there may be some overlap in participation between the survey and interviews. However, the open‐ended responses and prompt interview questions were different, and interviews allowed for more in‐depth responses. Future work could focus on medical staff needs and perspectives, and a broader range of health services, including those who do not have a focus of Aboriginal and Torres Strait Islander health and well‐being.

## 8 Conclusions

Overall, this work identifies clear policy and local service opportunities to strengthen primary healthcare to better serve Aboriginal and Torres Strait Islander children and adolescents; in particular those with complex needs stemming from trauma. In addressing these barriers and focussing on the identified enablers consistent with the WHO standards, there is opportunity to improve the health of Aboriginal and Torres Strait Islander children and adolescents, therefore, strengthening preventive and supportive care in the primary health sector.

## Author Contributions

Christianna Digenis: Data curation, formal analysis, investigation, project administration, validation, visualisation/data presentation, writing – original draft, writing – review and editing. Rachel Reilly: Data curation, formal analysis, investigation, project administration, validation, visualisation/data presentation, writing – original draft, writing – review and editing. Peter Azzopardi: Conceptualisation, formal analysis, funding acquisition, investigation, methodology, project administration, supervision, validation, writing – original draft, writing – review and editing. Hilina Winkenweder: Formal analysis, investigation, visualisation/data presentation, writing – review and editing. Odette Pearson: Conceptualisation, funding acquisition, methodology, writing – review and editing. Kane Ellis: Conceptualisation, project administration, supervision, writing – review and editing. Jane Fisher: Conceptualisation, funding acquisition, methodology, supervision, writing – review and editing. Debra J. Rickwood: Conceptualisation, funding acquisition, methodology, supervision, writing – review and editing. Choong‐Siew Yong: Conceptualisation, funding acquisition, methodology, writing – review and editing. Ngiare Brown: Conceptualisation, formal analysis, funding acquisition, investigation, methodology, project administration, supervision, validation, writing – original draft, writing – review and editing.

## Funding

This work was supported by the National Health and Medical Research Council (APP1201471); Ian Potter Foundation (22055).

## Disclosure

Not commissioned; externally peer reviewed.

## Conflicts of Interest

The authors declare no conflicts of interest.

## Supporting information


**Data S1:** mja270150‐sup‐0001‐supinfo.pdf.

## Data Availability

Data may be available upon reasonable request by contacting the principal researcher, Ngiare Brown.
