# Supplementary material for: Ngalaiya Boorai Gabara Budbut: A Qualitative Study With Primary Care Providers to Understand Perceived Needs, Enablers, Barriers and Opportunities to Strengthen Care
Source: Med J Aust. 2026 Mar 4;224(3):e70150. doi: 10.5694/mja2.70150 (PMC12958009; doi:10.5694/mja2.70150)
Supplement: Supplementary file 1 — Data S1: mja270150‐sup‐0001‐supinfo.pdf. [file MJA2-224-0-s001.pdf]

The Medical Journal of Australia

Supporting Information

Supplementary material

This appendix was part of the submitted manuscript and has been peer reviewed.
It is posted as supplied by the authors.

Appendix to: Digenis C, Reilly R, Azzopardi P, et al. Ngalaiya Boorai Gabara Budbut: A Qualitative Study with Primary Care Providers to Understand Perceived Needs, Enablers, Barriers and Opportunities to Strengthen Care. *Med J Aust* 2026; doi: 10.5694/mja2.70150.

Interview questions

Health issues for young people<br>I would like to learn a bit about your perspective of the health issues facing young people.<br>• From your perspective, what are the key health issues for young people?<br>• Prompts: Mental health, sexual and reproductive health, smoking, use of alcohol or drugs, healthy eating and physical activity<br>• How do you think this impacts a young person's life?<br>• Prompts: Other areas of life or well-being e.g. mental, social and emotional, school, work, family, friends, engaging in healthy life choices<br>• From your perspective, what are the major social and emotional well-being issues facing children and young people today?<br>• Prompts: Being away/disconnected from culture, family or friends, racism and discrimination, bullying and online harassment, climate change, etc.<br>• What does a young person need in their lives to remain healthy?<br>• Prompts: Supportive network, friends, family, school, work, active lifestyle, nutritious food, taking medications
Enablers and barriers<br>• In your opinion, what are the challenges and barriers to providing healthcare for young people?<br>• Prompts: knowledge of services, ability of services to cater for young people, suitable hours for young people, availability of services (i.e. limited mental health services)<br>• What supports and enables good healthcare to young people?<br>• Prompts: allocated resources, friendly and welcoming services, collaborative approaches<br>• What do you think could be done to improve access to health service for young people?<br>• Prompts: tailored service, welcoming environment, respect, young people included in the decision-making process
Service delivery<br>• How do you think that healthcare to young people can be improved?<br>• Prompts: training, finding out from young people, including young people in the service design or structure<br>• What would help you strengthen/enhance the healthcare you provide to young people?<br>• Prompts: training, support, leadership, funding, resources<br>• What areas of training would support you/would you like in healthcare provision for young people?<br>• Prompts: sexual health training, communication, rights, cultural safety<br>• What would an ideal youth friendly service look like?<br>• Prompts: welcoming to young people, young people represented in the service structure, services that are specific to young people's needs

Open-ended survey questions

From your perspective, what are the major health and well-being issues facing Aboriginal and Torres Strait Islander children today?
Is there any other training in child or adolescent health that you would like?
Trauma-informed, culturally safe care<br>What does 'trauma informed care' mean to your practice?
What does 'cultural safety' mean to your practice?

COREQ (COnsolidated criteria for REporting Qualitative research) Checklist

A checklist of items that should be included in reports of qualitative research. You must report the page number in your manuscript where you consider each of the items listed in this checklist. If you have not included this information, either revise your manuscript accordingly before submitting or note N/A.

Topic	Item No.	Guide Questions/Description	Reported on Page No.
**Domain 1: Research team and reflexivity**			
Personal characteristics			
Interviewer/facilitator	1	Which author/s conducted the interview or focus group?	3
Credentials	2	What were the researcher's credentials? E.g. PhD, MD	Title page
Occupation	3	What was their occupation at the time of the study?	3
Gender	4	Was the researcher male or female?	N/A
Experience and training	5	What experience or training did the researcher have?	3
Relationship with participants			
Relationship established	6	Was a relationship established prior to study commencement?	N/A
Participant knowledge of the interviewer	7	What did the participants know about the researcher? e.g. personal goals, reasons for doing the research	4
Interviewer characteristics	8	What characteristics were reported about the interviewer/facilitator? e.g. Bias, assumptions, reasons and interests in the research topic	3, 4
**Domain 2: Study design**			
Theoretical framework			
Methodological orientation and Theory	9	What methodological orientation was stated to underpin the study? e.g. grounded theory, discourse analysis, ethnography, phenomenology, content analysis	3
Participant selection			
Sampling	10	How were participants selected? e.g. purposive, convenience, consecutive, snowball	3
Method of approach	11	How were participants approached? e.g. face-to-face, telephone, mail, email	3
Sample size	12	How many participants were in the study?	4
Non-participation	13	How many people refused to participate or dropped out? Reasons?	N/A
Setting			
Setting of data collection	14	Where was the data collected? e.g. home, clinic, workplace	3
Presence of non-participants	15	Was anyone else present besides the participants and researchers?	3
Description of sample	16	What are the important characteristics of the sample? e.g. demographic data, date	4
Data collection			
Interview guide	17	Were questions, prompts, guides provided by the authors? Was it pilot tested?	17, 18, 19
Repeat interviews	18	Were repeat interviews carried out? If yes, how many?	N/A
Audio/visual recording	19	Did the research use audio or visual recording to collect the data?	3
Field notes	20	Were field notes made during and/or after the interview or focus group?	N/A
Duration	21	What was the duration of the interviews or focus group?	4
Data saturation	22	Was data saturation discussed?	N/A 3 representation discussed
Transcripts returned	23	Were transcripts returned to participants for comment and/or	N/A

Topic	Item No.	Guide Questions/Description	Reported on Page No.
		correction?	
**Domain 3: analysis and findings**			
Data analysis			
Number of data coders	24	How many data coders coded the data?	3
Description of the coding tree	25	Did authors provide a description of the coding tree?	N/A
Derivation of themes	26	Were themes identified in advance or derived from the data?	3
Software	27	What software, if applicable, was used to manage the data?	3
Participant checking	28	Did participants provide feedback on the findings?	N/A
Reporting			
Quotations presented	29	Were participant quotations presented to illustrate the themes/findings?<br>Was each quotation identified? e.g. participant number	Yes
Data and findings consistent	30	Was there consistency between the data presented and the findings?	Yes
Clarity of major themes	31	Were major themes clearly presented in the findings?	Yes
Clarity of minor themes	32	Is there a description of diverse cases or discussion of minor themes?	Yes

Developed from: Tong A, Sainsbury P, Craig J. Consolidated criteria for reporting qualitative research (COREQ): a 32-item checklist for interviews and focus groups. *International Journal for Quality in Health Care*. 2007. Volume 19, Number 6: pp. 349 – 357

Once you have completed this checklist, please save a copy and upload it as part of your submission. DO NOT include this checklist as part of the main manuscript document. It must be uploaded as a separate file.

CONSIDER statement

Item	Checklist Item	Section
Governance		
1.	Describe partnership agreements between the research institution and Indigenous-governing organization for the research, (e.g., Informal agreements through to MOU (Memorandum of Understanding) or MOA (Memorandum of Agreement)).	Yes- end of introduction
2.	Describe accountability and review mechanisms within the partnership agreement that addresses harm minimization.	Yes- Methods describe the governance group.
3.	Specify how the research partnership agreement includes protection of Indigenous intellectual property and knowledge arising from the research, including financial and intellectual benefits generated (e.g., development of traditional medicines for commercial purposes or supporting the Indigenous community to develop commercialization proposals generated from the research).	Yes-use of Aboriginal research ACCORD specified
Prioritization		
4.	Explain how the research aims emerged from priorities identified by either Indigenous stakeholders, governing bodies, funders, non-government organization(s), stakeholders, consumers, and empirical evidence	Yes – conclusion of background
Relationships (Indigenous stakeholders/participants and Research team)		
5.	Specify measures that adhere and honor Indigenous ethical guidelines, processes, and approvals for all relevant Indigenous stakeholders, recognizing that multiple Indigenous partners may be involved, e.g., Indigenous ethics committee approval, regional/national ethics approval processes.	Yes- approved by Indigenous ethics committee. Outlined in methods section
6.	Report how Indigenous stakeholders were involved in the research processes (i.e., research design, funding, implementation, analysis, dissemination/recruitment).	Yes- governance group and partner organisations described
7.	Describe the expertise of the research team in Indigenous health and research.	Yes- In methods section under research team, governance and reflexivity.
Methodologies		

8.	Describe the methodological approach of the research including a rationale of methods used and implication for Indigenous stakeholders, e.g., privacy and confidentiality (individual and collective)	Yes-Methods
9.	Describe how the research methodology incorporated consideration of the physical, social, economic and cultural environment of the participants and prospective participants. (e.g., impacts of colonization, racism, and social justice). As well as Indigenous worldviews.	Yes- Methods
Participation		
10.	Specify how individual and collective consent was sought to conduct future analysis on collected samples and data (e.g., additional secondary analyses; third-parties accessing samples (genetic, tissue, blood) for further analyses).	Yes-Methods
11.	Described how the resource demands (current and future) placed on Indigenous participants and communities involved in the research were identified and agreed upon including any resourcing for participation, knowledge, and expertise	Yes- Methods
12.	Specify how biological tissue and other samples including data were stored, explaining the processes of removal from traditional lands, if done, and of disposal.	N/A
Capacity		
13.	Explain how the research supported the development and maintenance of Indigenous research capacity (e.g., specific funding of Indigenous researchers).	Yes -discussion
14.	Discuss how the research team undertook professional development opportunities to develop the capacity to partner with Indigenous stakeholders?	Yes- discussion
Analysis and interpretation		
15.	Specify how the research analysis and reporting supported critical inquiry and a strength-based approach that was inclusive of Indigenous values.	Yes – Methods
Dissemination		
16.	Describe the dissemination of the research findings to relevant Indigenous governing bodies and peoples.	Yes- methods and discussion
17.	Discuss the process for knowledge translation and implementation to support Indigenous advancement (e.g., research capacity, policy, investment).	Yes-discussion
